# High levels of arbuscular mycorrhizal fungus colonization on *Medicago truncatula* reduces plant suitability as a host for pea aphids (*Acyrthosiphon pisum*)

**DOI:** 10.1111/1744-7917.12631

**Published:** 2018-09-17

**Authors:** Elisa Garzo, Eric Rizzo, Alberto Fereres, S. Karen Gomez

**Affiliations:** ^1^ Instituto de Ciencias Agrarias—Consejo Superior de Investigaciones Científicas (ICA‐CSIC) Madrid Spain; ^2^ School of Biological Sciences University of Northern Colorado Greeley Colorado USA

**Keywords:** Aphididae, arbuscular mycorrhizal fungus, electrical penetration graph (EPG), Fabaceae, Glomeraceae, insect–plant–microbe interactions, symbiosis

## Abstract

This study sheds light on a poorly understood area in insect–plant–microbe interactions, focusing on aphid probing and feeding behavior on plants with varying levels of arbuscular mycorrhizal (AM) fungus root colonization. It investigates a commonly occurring interaction of three species: pea aphid *Acyrthosiphon pisum*, barrel medic *Medicago truncatula*, and the AM fungus *Rhizophagus irregularis*, examining whether aphid‐feeding behavior changes when insects feed on plants at different levels of AM fungus colonization (42% and 84% root length colonized). Aphid probing and feeding behavior was monitored throughout 8 h of recording using the electrical penetration graph (EPG) technique, also, foliar nutrient content and plant growth were measured. Summarizing, aphids took longer to reach their 1st sustained phloem ingestion on the 84% AM plants than on the 42% AM plants or on controls. Less aphids showed phloem ingestion on the 84% AM plants relative to the 42% AM plants. Shoots of the 84% AM plants had higher percent carbon (43.7%) relative to controls (40.5%), and the 84% AM plants had reduced percent nitrogen (5.3%) relative to the 42% AM plants (6%). In conclusion, EPG and foliar nutrient data support the hypothesis that modifications in plant anatomy (e.g., thicker leaves), and poor food quality (reduced nitrogen) in the 84% AM plants contribute to reduced aphid success in locating phloem and ultimately to differences in phloem sap ingestion. This work suggests that *M. truncatula* plants benefit from AM symbiosis not only because of increased nutrient uptake but also because of reduced susceptibility to aphids.

## Introduction

Arbuscular mycorrhizal (AM) fungi are obligate biotrophs of the phylum Glomeromycota that form symbiosis with most vascular plants in a relationship dating back 450 million years (Remy *et al*., 1994). Through the AM symbiosis, plants receive nutrients such as phosphate (P), nitrogen (N), zinc, sulfur, copper, and other elements that are supplied by the AM fungus in exchange for carbon (C) (Francis & Read, 1984; Marschner & Dell, 1994; Smith & Read, 1997; Mader *et al*., 2000). It has been estimated that about 20% of C fixed from photosynthesis is transferred from the plant to the fungus (Harrison, 1999). In addition, mycorrhizal plants exhibit resistance to drought (Allen & Boosalis, 1983; Sieverding, 1986), salinity (Bothe, 2012; Porcel *et al*., 2012), and diseases (Cordier *et al*., 1998; Pozo *et al*., 2002; Liu *et al*., 2007; Fiorilli *et al*., 2011; Campos‐Soriano *et al*., 2012; Nair *et al*., 2015a,b; Bruisson *et al*., 2016; Delavaux *et al*., 2017; Wang *et al*., 2018).

An aspect of the symbiosis that has gained more attention in the last 15 years involves interactions between AM fungi, plants, and above‐ and below‐ground herbivores (Rasmann *et al*., 2017). Our research focuses on the evolving field of insect–plant–microbe interactions between AM fungi, plants, and phloem feeders (aphids). It has been proposed that mycorrhizal fungi could alter plant interactions with insects through multiple mechanisms involving changes in plant nutritional quantity and quality, defensive strategies and tolerance (Bennett *et al*., 2006). Moreover, AM fungi alter plant tolerance to insect herbivores and chemical defenses simultaneously (Tao *et al*., 2016). In milkweed (*Asclepias* spp.), tolerance increases with increased foliar P levels, whereas chemical defenses increase with increased foliar N levels. Chemical defenses can increase or decrease based on plant growth, which can be influenced by the AM symbiosis (Tao *et al*., 2016). The phytohormone jasmonic acid (JA) has been proposed as a major player in modulating plant defenses that lead to mycorrhiza‐induced resistance (MIR) (Jung *et al*., 2012), but there are still limited data supporting its role (Song *et al*., 2013).

The indirect effects of AM fungi on plant interactions with aphids has resulted in beneficial (Gange *et al*., 2002; Babikova *et al*., 2014a; Maurya *et al*., 2018; Meier & Hunter, 2018), detrimental (Guerrieri *et al*., 2004; Babikova *et al*., 2013; Maurya *et al*., 2018; Meier & Hunter, 2018), or no effects on aphids (Gehring & Bennett, 2009; Hartley & Gange, 2009; Karley *et al*., 2017). Previous research found a positive effect of the AM symbiosis on aphid adult weight (Gange & West, 1994; Gange *et al*., 1999), percent growth (Gange *et al*., 2002), and aphid abundance (Babikova *et al*., 2014a). Mycorrhizal broad bean (*Vicia faba* L.) plants release lower amounts of sesquiterpenes (e.g., (*Ε*)‐caryophyllene and (*E*)‐β‐farnesene) making these plants more attractive to pea aphids (*Acyrthosiphon pisum* Harris) (Babikova *et al*., 2014a). Pea aphids grew faster if plants were already colonized by AM fungi (40%–60% root length colonized [RLC]) at the onset of aphid feeding compared with controls. However, when plants were colonized with AM fungi (20%–40% RLC) after aphid infestation, pea aphids grew slower compared with controls (Babikova *et al*., 2014a). It seems that P does not play much of a role in pea aphid attraction to mycorrhizal broad bean plants (Babikova *et al*., 2014b). Interestingly, the green peach aphid's (*Myzus persicae* Sulzer) relative growth rate was reduced when insects fed on young *Plantago lanceolata* L. plants with a less established AM symbiosis (10% RLC), whereas aphids benefited by feeding on older plants with a well‐established AM symbiosis (80% RLC) (Tomczak & Müller, 2017). However, most first‐ and second‐generation nymphs reared on nonmycorrhizal plants were heavier compared to those reared on young and old mycorrhizal plants (Tomczak & Müller, 2017). Therefore, previous work demonstrates a diverse range of effects of AM symbiosis on aphid performance, but with clear examples of different physiological events impacting plant host suitability.

Aphid feeding behavioral studies could help us better understand why aphids benefit or are disadvantaged when feeding on plants that have different levels of AM fungus root colonization. So far, a few aphid behavioral traits (activity, exploration, and boldness) were examined on two plant species that were either noncolonized or colonized by AM fungi (Tomczak *et al*., 2016). It was shown recently that extrinsic (host plant quality modulated by AM symbiosis) and intrinsic (aphid age) variables can impact aphid performance and behavior (activity and exploration) differently (Tomczak & Müller, 2018). Aphids are known to ingest all compounds present in sieve elements such as carbohydrates, amino acids, proteins, water, and others. Probing and feeding behavior of aphids is not directly observable, but can be monitored using the electrical penetration graph (EPG) technique (Tjallingii, 1985; Tjallingii *et al*., 2010). The EPG technique has been widely used to investigate different aspects of insect–plant interactions. For example, EPG was used to characterize host plant resistance to hemipterans (Vanhelden & Tjallingii, 1993; Alvarez *et al*., 2013) to understand the transmission mechanisms of plant pathogens by their insect vectors (Fereres & Moreno, 2009; Moreno *et al*., 2012), to understand the mode of action of pesticides (Harrewijn & Kayser, 1997; Garzo *et al*., 2016), and to examine the feeding behavior of aphids on plants colonized by fungal endophytes (Bastias *et al*., 2017). Surprisingly, few studies used the EPG technique to examine whether the feeding behavior of aphids changes during feeding on mycorrhizal plants. It was shown recently that aphids fed longer, and spent a larger proportion of the overall time within the phloem of mycorrhizal plants (26.7% RLC) (Simon *et al*., 2017). It was proposed that the changes in aphid feeding behavior were probably associated with increased vascular bundle size in mycorrhizal plants (Simon *et al*., 2017). However, to our knowledge, there is limited information about aphid feeding behavior on plants of the same genotype and age that exhibit different levels of AM fungus colonization.

Here, we applied the EPG technique to gain more information on aphid probing and feeding behavior on mycorrhizal plants. We specifically monitored the impact of the AM symbiosis, using plants of the same age, on the feeding behavior of aphids. Our biological system included the pea aphid, *A. pisum*, the model plant *M. truncatula* Gaertn., and the generalist AM fungus *Rhizophagus irregularis* (Blaszk., Wubet, Renker, and Buscot) C. Walker and A. Schüßler (formerly *Glomus intraradices*). Pea aphids are distributed around the world, they feed on legumes including *M. truncatula*, and they are closely related to the green peach aphid (*M. persicae*) and the Russian wheat aphid (*Diuraphis noxia* Kurdjumov) (International Aphid Genomics Consortium, 2010). In the present study, we investigate whether pea aphid feeding behavior changes when insects feed on plants at different levels of AM fungus colonization. Additionally, we measured foliar nutrient levels and plant growth parameters on control and mycorrhizal plants.

## Materials and methods

### Plant growth conditions

Three groups of 60 *Medicago truncatula* plants line A17, reported as aphid‐susceptible (Gao *et al*., 2007), were grown staggered. Seeds were scarified and surface‐sterilized as previously described (Liu *et al*., 2007). Soil (Kekkilä 50/50 provided by Projar, S.A., Madrid, Spain) was autoclaved three times (120 °C, 15 psi, 1 h) and river sand was washed and autoclaved once (120 °C, 15 psi, 1 h). Pots (12 cm in diameter) were filled with one part of soil mixed with eight parts of sand. After transplanting, all plants were grown in a walk‐in growth chamber at 24 °C (day) and 20 °C (night), a photoperiod of 16 h, and light intensity of 200 µmol/m^2^/s. Plants were fertilized with 50 mL of ½ strength modified Hoagland's solution (100 µmol/L P [reduced], 15 mmol/L N [high]) twice per week, and watered with 50 mL of autoclaved deionized water every other day.

### Pea aphid rearing

Pea aphids (*Acyrthosiphon pisum*) lineage LSR1 were kindly provided by Dr. David Martínez (Instituto Cavanilles de Biodiversitat I Biología Evolutiva, Universidad de Valencia, Spain). A colony of aphids were reared on the universal host faba bean (*Vicia faba*) in growth chambers at 23 °C (day) and 18 °C (night), a photoperiod of 14 h, and light intensity of 200 µmol/m^2^/s. Nine‐ to ten‐day old apterous adult aphids were used for the EPG recordings.

### Arbuscular mycorrhizal fungus inoculation

Our goal was to achieve two distinct levels of AM fungus colonization on mycorrhizal plants to compare with nonmycorrhizal control plants. We aimed for a low to intermediate AM fungus colonization level that was used in published studies (15%–55% RLC) (Gange *et al*., 1999; Hempel *et al*., 2009; Koricheva *et al*., 2009; Babikova *et al*., 2014a; Simon *et al*., 2017; Maurya *et al*., 2018), and a higher level of AM fungus colonization (>55% RLC) (Tomczak & Müller, 2017; Maurya *et al*., 2018). A commercially available spore suspension of *R. irregularis* DAOM197198 (Mycovitro S.L., Granada, Spain) containing 450 spores/mL was used for the experiment. Roots of 24‐d‐old (postseed sterilization) *M. truncatula* plants were inoculated as follows: 5 mL per pot of suspension without spores (referred as nonmycorrhizal control), 3 mL per pot of suspension without spores + 2 mL per pot of spore suspension (∼900 spores), and 5 mL per pot of spore suspension (∼2250 spores). To assess root colonization levels prior to EPG recordings, three plants (5 weeks postinoculation) from each colonization level were harvested and roots were ink‐stained as previously described (Vierheilig *et al*., 1998).

### Assessment of probing and feeding behavior of aphids on plants with different levels of AM fungus colonization

To determine whether the pea aphid feeding behavior changes when insects feed on nonmycorrhizal and mycorrhizal plants, we compared the following treatments: (1) nonmycorrhizal control; (2) low to intermediate level of AM fungus colonization; and (3) high level of AM fungus colonization. Feeding behavior experiments were conducted on *M. truncatula* plants at 6–7 weeks postinoculation for each treatment. Thirty‐five plants per treatment were used for recordings. Every plant per treatment received a single pea aphid and was considered a biological replicate. Figure S1 shows a schematic representation of the experimental set up described in this section. First, apterous adult aphids were immobilized by a vacuum device, and a gold wire (20 mm length, 18.5 µm diameter; EPG Systems, Wageningen, the Netherlands) was gently attached to the aphid dorsum using hand‐mixed, water‐based silver conductive paint glue (EPG Systems). The opposite end of the gold wire was glued with a droplet of paint to a copper extension wire (20 mm in length) which was inserted into the input of the EPG head stage amplifier. Another copper electrode (100 mm length, 2 mm diameter) was inserted into the soil of the plant container to close the circuit. Aphids were starved for approximately 1 h for the acclimatization period between the time of wiring and the beginning of EPG recording. A single aphid per plant was placed on a fully expanded trifoliolate leaf (Fig. S1C). Aphids were allowed to probe and feed on plants for 8 h once connected to the EPG device. One Giga‐4 and one Giga‐8 DC‐EPG device with 1 GΩ (EPG Systems) were used to monitor the probing and feeding activities of aphids on *M. truncatula* plants. A USB analog/digital converter card (DI‐158U; DATAQ Instruments, Akron, OH, USA) was used to transfer the EPG signals to a laptop computer. EPG signals were acquired and analyzed using Stylet+ (EPG Systems) software for Windows.

### Analysis of electrical penetration graph waveforms

**Figure 1:**
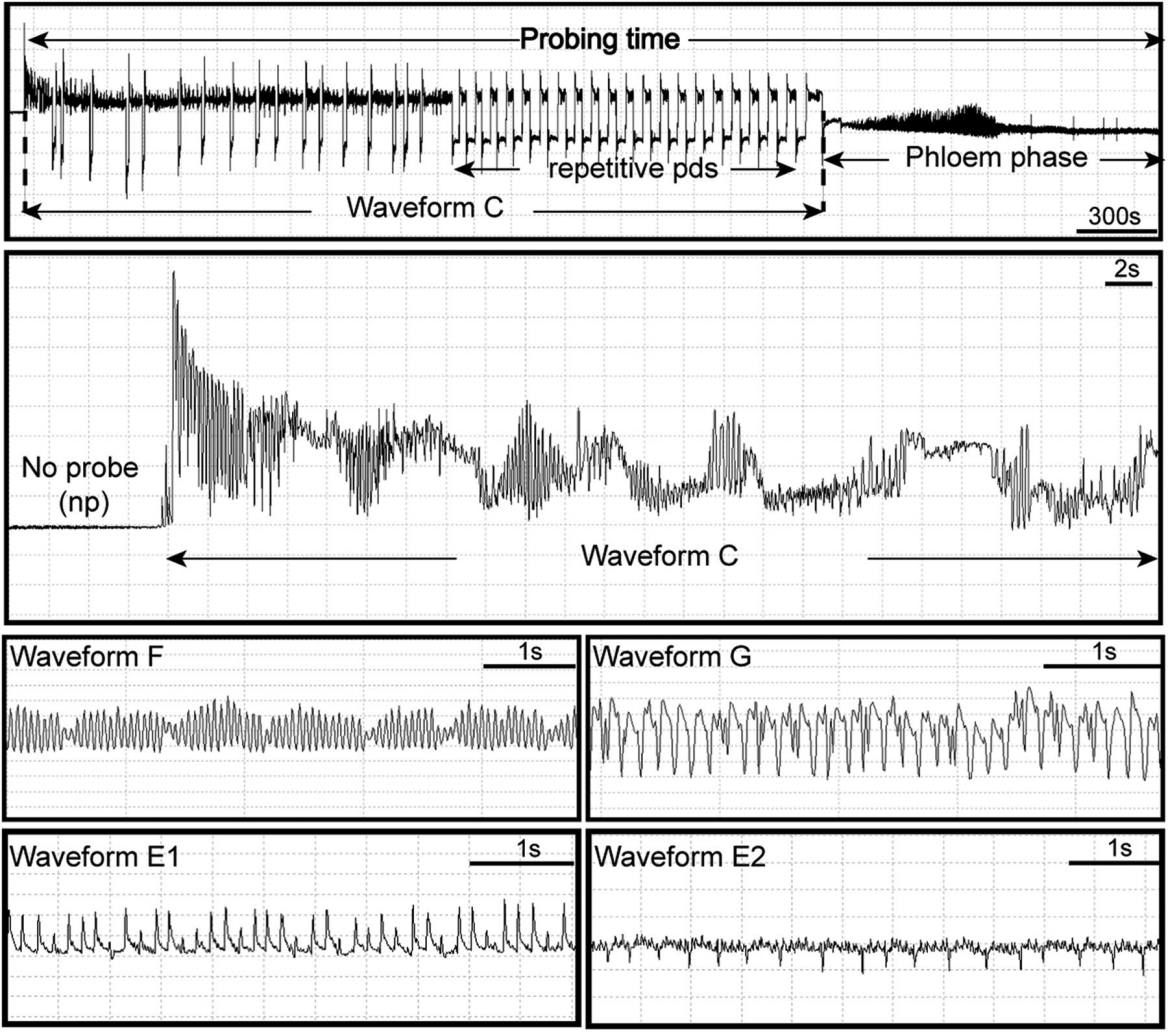
Electrical penetration graph (EPG) recording of pea aphids (*Acyrthosiphon pisum*) on *Medicago truncatula* plants. Waveform “np” represents nonprobing behavior (no stylet contact with the leaf tissue). Probing time represents the insertion of the stylet into the plant and performance of all subsequent behaviors within the plant including salivation, ingestion and stylet movements. Waveform “C” represents the intercellular apoplastic stylet pathway where the insect shows a cyclic activity of mechanical stylet penetration and secretion of saliva. Waveform “pd” represents intracellular punctures. Some species of aphids such as *A. pisum* show repetitive “pds” before the phloem salivation (E1) phase (Tjallingii & Gabrys, 1999). Waveform “F” represents derailed stylet mechanics and waveform “G” represents active intake of xylem sap. Two waveforms related to phloem phase: waveform “E1” represents salivation into phloem sieve elements at the beginning of the phloem phase, and waveform “E2” is correlated with passive phloem sap uptake from the sieve element.

EPG variables of 15 recordings per treatment were processed using the EPG‐Excel Data Workbook version 5.0 developed by Dr. Fereres’ team (Sarria *et al*., 2009). Recordings in which aphids exhibited aberrant behavior (no feeding during the first hour) were discarded. Figure 1 illustrates the EPG waveforms associated with specific stylet tip positions and activities when aphids probe and feed on plants (Kimmins & Tjallingii, 1985; Tjallingii & Esch, 1985; Tjallingii & Gabrys, 1999). Waveform “np” represents nonprobing behavior (no stylet contact with the leaf tissue), and waveform “C” represents the intercellular apoplastic stylet pathway where the insects show a cyclic activity of mechanical stylet penetration and secretion of saliva. Two waveforms related to phloem activity were recorded: waveform “E1,” which represents salivation into phloem sieve elements at the beginning of the phloem phase, and waveform “E2,” which is correlated with passive phloem sap uptake from the sieve element. Furthermore, waveform “G” represents active intake of xylem sap, and waveform “F” represents derailed stylet mechanics.

To analyze the impact of the AM symbiosis on insect probing and feeding behavior, a specific set of EPG variables were calculated as previously described (Backus *et al*., 2007). For example, the number of waveform events per insect (NWEI) was calculated using the sum of the number of events of a particular waveform divided by the total number of insects under each treatment, and the waveform duration per insect (WDI) was calculated using the sum of durations of each event of a particular waveform made by each individual insect that produced that waveform divided by the total number of insects under each treatment. The waveform duration per event (WDE) is the sum of the duration of the events for a particular waveform divided by the total number of events of that particular waveform under each treatment. The output generated with the EPG‐Excel Data Workbook (Sarria *et al*., 2009) for each given aphid (replicate) was used to calculate the treatment mean for each variable.

### Plant biomass, foliar nutrients, and AM fungus colonization

Plants were harvested following EPG recordings. Separate weights of root and shoot fresh tissue were determined as well as root length. Shoot tissue was placed in a drying oven (Heraeus D‐6450 Hanau) at 80 °C for 72 h to obtain dry weight and for elements content analysis. The entire root system was collected and ink‐stained to assess AM fungus colonization levels via the gridline–intersection method (McGonigle *et al*., 1990; Vierheilig *et al*., 1998). At least a 100 intersects per plant was used for AM fungus colonization quantification. Nonmycorrhizal plants did not show staining of fungal structures. After confirmation of AM fungus root colonization levels, plants were assigned into three groups: (1) control (nonmycorrhizal plants), (2) low to intermediate AM fungus colonization (mean: 42%; range: 23%–50% RLC), and (3) high AM fungus colonization (mean: 84%; range: 56%–100% RLC) (Table 1). Prior to analyzing EPG data, the AM fungus colonization level was determined for every plant in order to assign a plant to the final RLC range.

**Table 1:** Plant growth parameters of *Medicago truncatula* plants at different levels of arbuscular mycorrhizal (AM) fungus colonization: nonmycorrhizal control plants, 42% AM fungus‐colonized plants (range: 23%–50% RLC), and 84% AM fungus‐colonized plants (range: 56%–100% RLC). FW: fresh weight; DW: dry weight; L: length; RLC: root length colonized. Values represent means ± SE

	Control (*N* = 15)	42% AM (*N* = 15)	84% AM (*N* = 15)	*P*
Shoot FW (g)	3.9 ± 0.4 a	3.6 ± 0.5 a	4.3 ± 0.6 a	0.618
Shoot DW (g)	0.7 ± 0.1 a	0.6 ± 0.1 a	0.7 ± 0.1 a	0.611
Root FW (g)	3.1 ± 0.2 a	2.5 ± 0.1 b	3.2 ± 0.2 a	0.032*
Shoot FW/root FW	1.3 ± 0.2 a	1.4 ± 0.2 a	1.3 ± 0.1 a	0.853
Root L (cm)	25.3 ± 0.5 a	26.1 ± 0.9 a	26.4 ± 1.0 a	0.625
Colonization (%RLC)†	–	42.0 ± 2.1 a	84.2 ± 4.5 b	<0.001*

^*^Statistically significant *P* values (*P* ≤ 0.05) based on the Kruskal–Wallis test. Different letters indicate significant differences among treatments according to the Dwass–Steel–Critchlow–Fligner test for specific pairwise comparisons.

^†^ *P* value shown based on the Mann Whitney *U* test.

Organic carbon, total nitrogen, and solid organic matter from shoots were determined by dry combustion using a Thermo Flash 2000 NC Soil Analyzer. Phosphorus was extracted with calcium carbonate following Burriel and Hernando's method (Burriel & Hernando, 1950), and was quantified using an inductively coupled plasma‐atomic emission spectrometer (ICP‐OES) (Perkin Elmer Optima 4300 DV).

### Statistical analyses

Statistical analyses were performed using JMP 10.0 Pro Statistics software (SAS Institute, Cary, NC, USA). Raw data were checked for normality and homogeneity of variance using Shapiro–Wilk *W* test before performing the parametric tests. The data were analyzed by one‐way ANOVA test (after appropriate data transformation) for Gaussian distribution and the means were subsequently separated using the least significant difference (LSD) test. The Kruskal–Wallis test followed by the Dwass–Steel–Critchlow–Fligner test for specific pairwise comparisons and Mann–Whitney *U* test were performed for non‐Gaussian distribution. Percentage of aphids that showed a specific behavior in 30‐min intervals over a total recording time of 8 h was compared among the different treatment groups using the Chi‐square test. If the expected values were lower than 5, the Fisher's Exact Test was used. This analysis was done using Statview 4.0 software for Macintosh (Abacus Concepts, Berkeley, CA, USA). Significance was declared at *P* ≤ 0.05 for all variables.

## Results

### Effect of AM symbiosis on plant growth and foliar nutrient content

We hypothesized that high AM fungus‐colonized plants would show higher levels of leaf nutrients (e.g., phosphorous and nitrogen) relative to nonmycorrhizal plants. Overall, we found no statistically significant effect of the AM symbiosis on *M. truncatula* growth, except for root fresh weight (Table 1). There were no statistically significant differences between treatments for shoot fresh and dry weights, root length, and shoot to root fresh weight ratio (Table 1). Measurement of AM fungus colonization levels confirmed that inoculation with varying spore concentrations resulted in significant differences in root‐length colonized (RLC) (Table 1), and treatments were thereafter designated as the resulting RLC levels, 42% AM fungus‐colonized plants and 84% AM fungus‐colonized plants. In terms of foliar nutrients, we found differences among treatments for foliar carbon (C), nitrogen (N), and C/N (Table 2). There were no differences among treatments for phosphorus (P) and N/P (Table 2). Percent C was higher in shoots of the 84% AM fungus‐colonized plants (43.7% C) relative to control plants (40.5% C), while percent C in shoots of the 42% AM fungus‐colonized plants (42.6% C) was not different from the other treatments. Percent N was higher in shoots of the 42% AM fungus‐colonized plants (6% N) relative to the 84% AM fungus‐colonized plants (5.3% N), while percent N in shoots of control plants (5.4% N) was not different from the other treatments. Shoots of the 84% AM fungus‐colonized plants had higher C/N than shoots of the 42% AM fungus‐colonized plants. The C/N in shoots of control plants was not different from the other treatments.

**Table 2:** Foliar nutrient concentration and element ratios from *Medicago truncatula* plants at different levels of arbuscular mycorrhizal (AM) fungus colonization: nonmycorrhizal control plants, 42% AM fungus‐colonized plants (range: 23%–50% RLC), and 84% AM fungus‐colonized plants (range: 56%–100% RLC). RLC: root length colonized. Values represent means ± SE of 15 biological replicates

	Control	42% AM	84% AM	*P*
% C	40.5 ± 0.6 b	42.6 ± 0.6 ab	43.7 ± 0.6 a	0.0027*
% N	5.4 ± 0.2 ab	6.0 ± 0.2 a	5.3 ± 0.2 b	0.0431*
% P	0.2 ± 0.0 a	0.3 ± 0.0 a	0.3 ± 0.0 a	0.4609
C/N	7.6 ± 0.3 ab	7.3 ± 0.3 b	8.4 ± 0.3 a	0.0448*
N/P	27.8 ± 3.3 a	23.0 ± 1.6 a	21.6 ± 1.5 a	0.3174

^*^Statistically significant *P* values (*P* ≤ 0.05) based on one‐way ANOVA. Different letters indicate significant differences among treatments based on the least significant difference (LSD) test.

### Probing and feeding behavior of pea aphids on plants with different AM fungus colonization levels

Prior to performing EPG analyses, experimental plants were assigned into three groups based on the level of AM fungus root colonization: (1) control plants (0% RLC), (2) 42% AM fungus‐colonized plants, and (3) 84% AM fungus‐colonized plants (Table 1). We hypothesized that pea aphids would show a distinct pattern of probing and feeding behavior while feeding on mycorrhizal plants relative to nonmycorrhizal control plants.

The duration of the intercellular apoplastic stylet pathway “C” was higher in insects that fed on the 42% AM fungus‐colonized plants and/or the 84% AM fungus‐colonized plants relative to the control plants (Table 3, variable C, WDE). The time from the beginning of that probe to 1st E2 (phloem ingestion) and time from beginning of that probe to first sustained E2 were longer for aphids on the 84% AM fungus‐colonized plants than for aphids on the control plants (Table 3; 44.5 ± 14.4 min vs. 21.3 ± 1.9 min, *P* = 0.0396; and 56.1 ± 17.1 min vs. 24.3 ± 2.2 min, *P* = 0.0043, respectively). Likewise, pea aphids showed a reduced total duration (WDI) of sustained phloem sap ingestion (E2 > 10 min) on the 84% AM fungus‐colonized plants than on the 42% AM fungus‐colonized plants (Table 3; 45.0 ± 14.9 min vs. 147.3 ± 33.8 min, *P* = 0.0470). In addition, the time from the beginning of that probe to 1st sustained E2 (>10 min) increased when aphids fed on the 84% AM fungus‐colonized plants compared to the 42% AM fungus‐colonized plants and the control plants (Table 3; 56.1 ± 1.7, 26.5 ± 2.8, and 24.3 ± 2.2 min, *P* = 0.0043, respectively).

**Table 3:** Electrical penetration graph (EPG) variables describing the probing behavior of pea aphids (*Acyrthosiphon pisum*), during 8 h of recording, on *Medicago truncatula* plants with three levels of arbuscular mycorrhizal (AM) fungus colonization: nonmycorrhizal control plants, 42% AM fungus‐colonized plants (range: 23%–50% RLC), and 84% AM fungus‐colonized plants (range: 56%–100% RLC). RLC: root length colonized; PPW: proportion of individuals that produced the waveform type; NWEI: number of waveform event per insect; WDI: waveform duration (min) per insect; WDE: waveform duration (min) per event. Waveform np, nonprobing; waveform C, intercellular apoplastic stylet pathway; waveform E1, salivation into phloem sieve elements; waveform E2, passive phloem sap uptake from the sieve elements; sE2, sustained phloem sap ingestion (>10 min)

		Treatment			
Nonsequential variable		control	42% AM	84% AM	*P*
Nonprobe	PPW	15/15	15/15	15/15	
	NWEI	14.5 ± 2.5	12.9 ± 1.2	17.5 ± 2.3	0.3342
	WDI	198.8 ± 26.2	128.5 ± 18.4	173.5 ± 23.6	0.1026
	WDE	13.7 ± 1.8	10.0 ± 1.2	9.9 ± 0.9	0.7708
Probe	PPW	15/15	15/15	15/15	
	NWEI	14.3 ± 2.5	12.7 ± 1.1	17.2 ± 2.3	0.3504
	WDI	281.2 ± 26.2	351.5 ± 18.4	302.6 ± 23.3	0.0953
	WDE	19.7 ± 3.2	27.6 ± 4.5	17.6 ± 2.2	0.3912
C	PPW	15/15	15/15	15/15	
	NWEI	16.4 ± 2.6	14.7 ± 1.2	20.3 ± 2.1	0.1913
	WDI	129.6 ± 13.7	131.3 ± 15.2	161.8 ± 17.4	0.2663
	WDE	5.5 ± 0.4a	8.9 ± 0.7b	8.0 ± 0.5b	0.0017*
E1	PPW	11/15	12/15	9/15	
	NWEI	1.1 ± 0.3	1.4 ± 0.3	1.1 ± 0.3	0.4127
	WDI	2.7 ± 1.1	3.5 ± 1.1	1.9 ± 0.6	0.4713
	WDE	2.5 ± 1.1	2.5 ± 0.7	1.8 ± 0.4	0.2260
E2	PPW	10/15	12/15	9/15	
	NWEI	0.8 ± 0.2	1.3 ± 0.3	0.9 ± 0.2	0.2057
	WDI	96.4 ± 31.9	148.4 ± 33.6	46.5 ± 14.9	0.1034
	WDE	120.5 ± 36.6	111.3 ± 25.2	53.6 ± 9.4	0.4266
Longest E2	PPW	10/15	12/15	9/15	
	NWEI	–	–	–	–
	WDI	159.9 ± 41.0	165.1 ± 33.9	59.3 ± 11.8	0.0690
	WDE	–	–	–	–
sE2	PPW	9/15	11/15	7/15	
	NWEI	0.6 ± 0.1	1.1 ± 0.2	0.7 ± 0.2	0.2680
	WDI	95.9 ± 31.8ab	147.3 ± 33.8b	45.0 ± 14.9a	0.0470*
	WDE	159.9 ± 32.7	138.1 ± 24.6	67.5 ± 31.2	0.1526

Sequential variable					
					
Time to 1st probe from start of EPG	PPW	15/15	15/15	15/15	
	WDI	30.3 ± 8.4	19.3 ± 7.4	24.9 ± 7.3	0.3845
Time from 1st probe to 1st E2	PPW	11/15	12/15	9/15	
	WDI	264.9 ± 39.9	277.0 ± 35.3	329.4 ± 35.6	0.3775
Time from the beginning of that probe to 1st E2	PPW	11/15	12/15	9/15	
	WDI	21.3 ± 1.9a	29.3 ± 4.2ab	44.5 ± 14.4b	0.0396*
Time from 1st probe to 1st sustained E2 (>10 min)	PPW	9/15	11/15	7/15	
	WDI	307.2 ± 36.3	285.4 ± 37.0	367.8 ± 31.9	0.2402
Time from the beginning of that probe to 1st sustained E2 (>10 min)	PPW	9/15	11/15	7/15	
	WDI	24.3 ± 2.2a	26.5 ± 2.8a	56.1 ± 17.1b	0.0043*

^*^Statistically significant *P* values (*P* ≤ 0.05) based on one‐way ANOVA or the Kruskal–Wallis test. Values represent mean ± SE. Means within a row followed by different letters indicate significance difference among treatments based on the least significant difference (LSD) test or the Dwass–Steel–Critchlow–Fligner test for specific pairwise comparisons (non‐Gaussian distribution).

**Figure 2:**
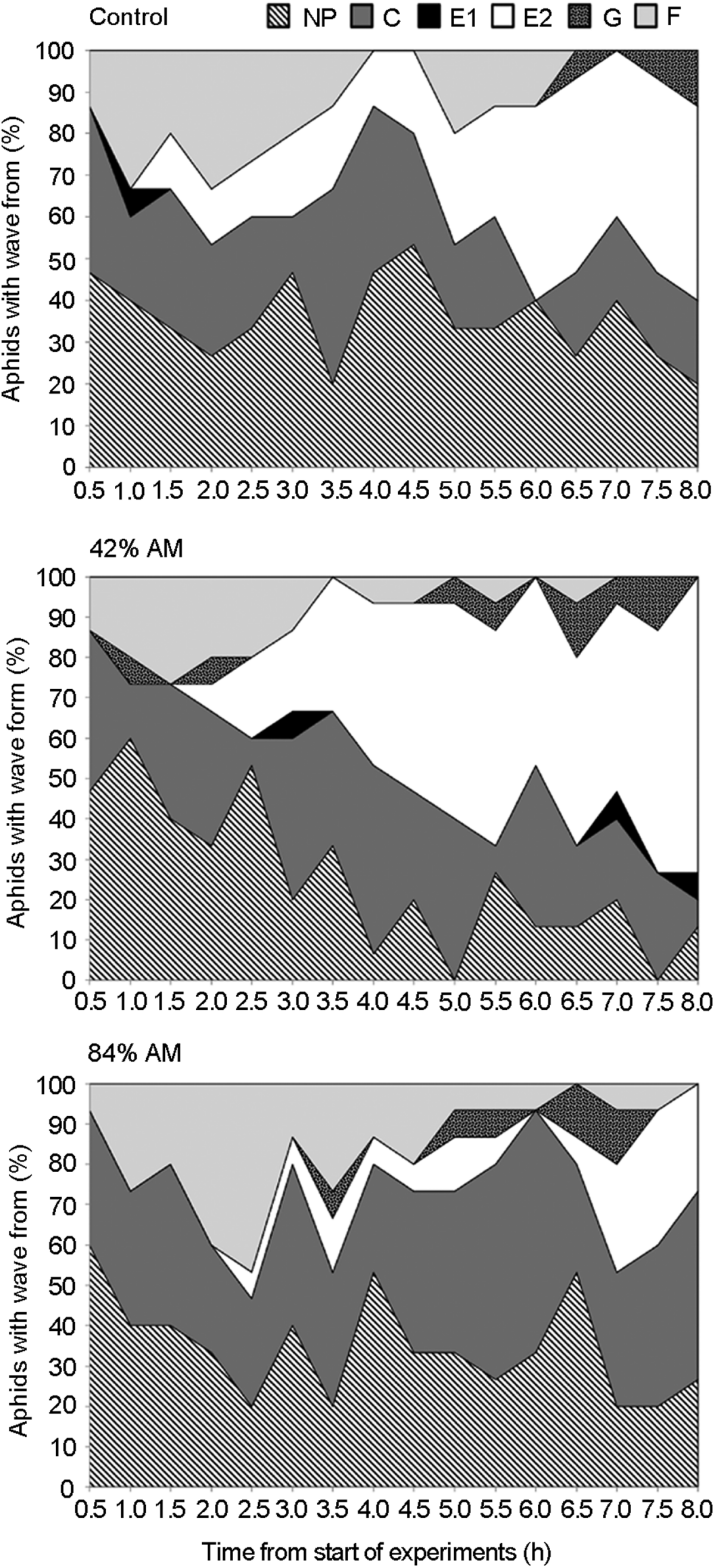
Percentage of pea aphids (*Acyrthosiphon pisum*) exhibiting specific electrical penetration graph (EPG) waveforms in 30‐min intervals over a total recording time of 8 h on *Medicago truncatula* plants with different levels of arbuscular mycorrhizal (AM) fungus colonization: nonmycorrhizal control plants, 42% AM fungus‐colonized plants (range: 23%–50% RLC), and 84% AM fungus‐colonized plants (range: 56%–100% RLC). RLC = root length colonized. Waveform np, nonprobing; waveform C, intercellular apoplastic stylet pathway; waveform F, derailed stylet mechanics; G, active xylem ingestion; waveform E1, salivation into phloem sieve elements; waveform E2, passive phloem sap uptake from the sieve elements.

The temporal evolution that show a specific feeding behavior in 30‐min intervals over a total recording time of 8 h of pea aphids on *M. truncatula* are represented in Figure 2. During the initial hours (1st–3rd hours), the feeding behavior was similar in all treatments as pea aphids exhibited pathway activities (waveform C) and nonprobing activities (waveform np). However, during the 4th and subsequent hours (5th, 6th, and 8th), we observed a higher percentage of aphids showing phloem ingestion (waveform E2) on the 42% AM fungus‐colonized plants than on the 84% AM fungus‐colonized plants. After 8 h of recording, 73% of aphids showed phloem ingestion (waveform E2) on the 42% AM fungus‐colonized plants, whereas 26.7% of aphids showed phloem ingestion on the 84% AM fungus‐colonized plants (Fig. 2; *χ* ^2^ = 6.533; df = 2; *P* = 0.0106). Overall, no differences were observed between the percentages of aphids showing phloem ingestion (E2) on control plants versus mycorrhizal plants. Except during the 6th hour, none of the aphids showed phloem ingestion on the 84% AM fungus‐colonized plants compared to aphids on the control plants (Fig. 2; 0% vs. 46.7% of aphids showed phloem ingestion, respectively).

**Figure 3:**
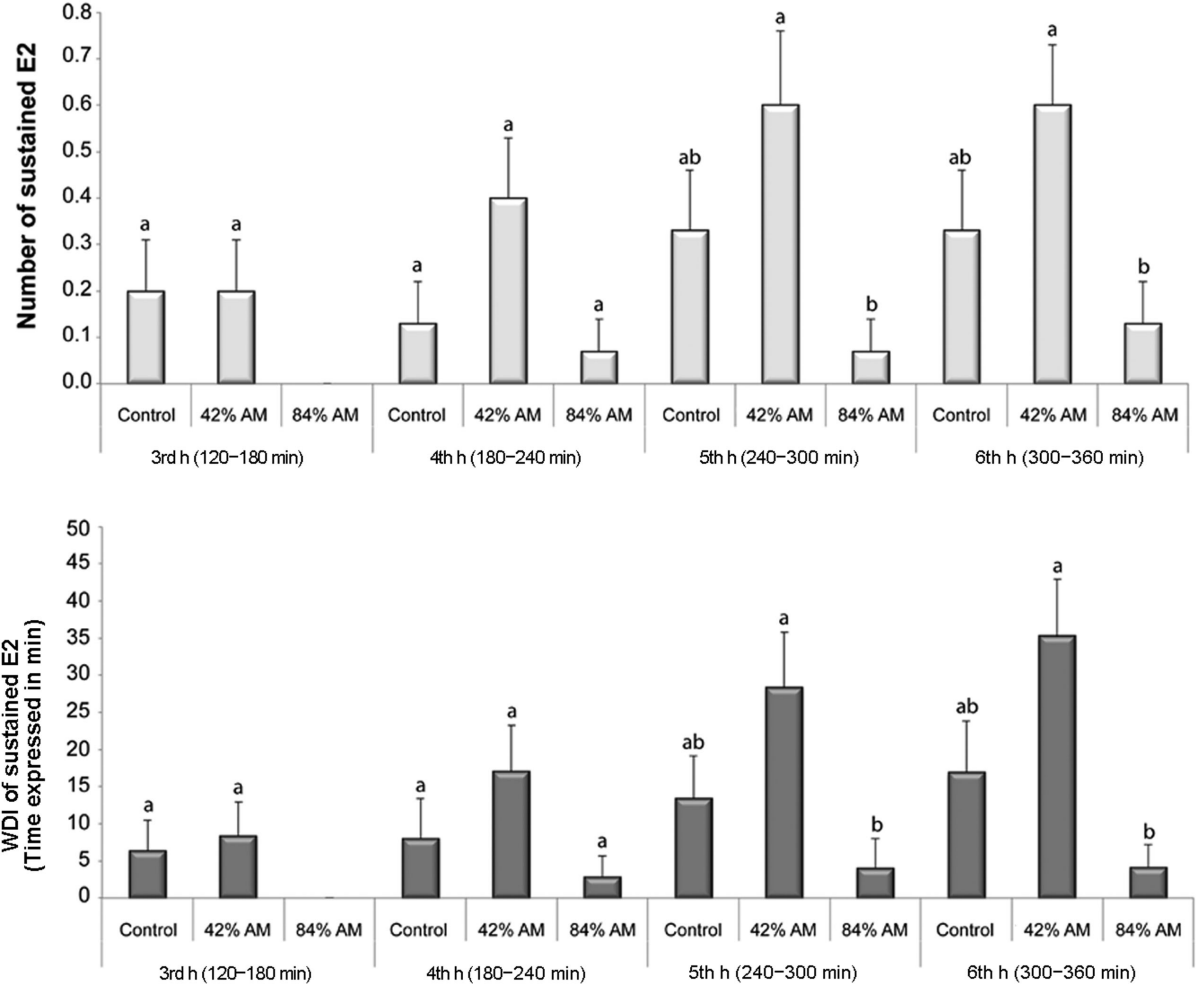
Analysis of probing and feeding behavior of pea aphids (*Acyrthosiphon pisum*) on *Medicago truncatula* plants with different levels of arbuscular mycorrhizal (AM) fungus colonization: nonmycorrhizal control plants, 42% AM fungus‐colonized plants (range: 23%–50% RLC), and 84% AM fungus‐colonized plants (range: 56%–100% RLC). The analysis represents the 3rd (120–180 min), 4th (180–240 min), 5th (240–300 min), and 6th (300–360 min) hour of recording. RLC = root length colonized. NWEI, number of waveform events per insect; WDI, waveform duration per insect. Different letters indicate significant differences among treatments within a feeding period based on the Kruskal–Wallis test followed by the Dwass–Steel–Critchlow–Fligner test for specific pairwise comparisons.

When focusing on phloem‐related EPG variables at varying time intervals (120–180, 180–240, 240–300, and 300–360 min) (Fig. 3), we detected differences during the 240–300 and 300–360 min time intervals. We found that the number of times aphids were able to ingest phloem sap (number of waveform events per insect [NWEI]), and the waveform duration per insect (WDI) of the sustained phloem ingestion (sE2) were reduced when aphids fed on the 84% AM fungus‐colonized plants compared to those that fed on the 42% AM fungus‐colonized plants (Fig. 3).

## Discussion

To the extent of our knowledge, this is the first report studying the outcome of insect–plant–microbe interactions with a focus on phloem feeders’ probing and feeding behavior on a leguminous plant with different levels of root colonization by AM fungi. In general, positive effects of the AM symbiosis on phloem feeders have been documented (Gange *et al*., 1999; Hempel *et al*., 2009; Koricheva *et al*., 2009; Babikova *et al*., 2014a; Simon *et al*., 2017; Maurya *et al*., 2018; Meier & Hunter, 2018). In previous studies, the extent of root colonization by AM fungi ranged between 15% and 55% RLC. A recent study reported that the aphid *Sitobion avenae* showed more sustained phloem ingestion (sE2) phases, which lasted longer than 10 min during feeding on aphid‐resistant wheat (*Triticum monococcum*) colonized by AM fungi (26.7% RLC) (Simon *et al*., 2017). In the present study, pea aphids spent more time in sE2 phase on *M. truncatula* plants with 42% AM fungus colonization levels compared to the 84% AM fungus‐colonized plants (Table 1, Fig. 3). Nevertheless, the impact of high levels of AM fungus root colonization on aphid–plant interactions is mostly unknown. We expected aphids to show difficulties finding and reaching the phloem (e.g., sieve elements), especially on high AM fungus‐colonized plants. This prediction was supported by previous studies showing anatomical modifications (e.g., leaf thickness) in mycorrhizal plants (Krishna *et al*., 1981; Simon *et al*., 2017). Interestingly, the data support our hypothesis indicating that the pea aphid feeding behavior was negatively affected during feeding on the 84% AM fungus‐colonized plants. We found that the aphid feeding efficiency (ability to find the phloem sieve elements in the successful probing) was significantly reduced during feeding on the 84% AM fungus‐colonized plants, indicating that aphids needed more time to reach their 1st sE2 phase compared to those insects feeding on the 42% AM fungus‐colonized plants (56.1 ± 17.1 and 26.5 ± 2.8 min, respectively) or on control plants (56.1 ± 17.1, 26.5 ± 2.8, and 24.3 ± 2.2 min, respectively).

When green peach aphid performance on young and old, nonmycorrhizal and mycorrhizal plants was examined, it was found that the body mass of first‐ and second‐generation nymphs decreased when insects were reared on older mycorrhizal plants (80% RLC) compared to nonmycorrhizal plants (Tomczak & Müller, 2017). It was proposed that a decreased food quality in older mycorrhizal plants caused negative effects on aphid body mass (Tomczak & Müller, 2017). Interestingly, we obtained similar results using plants of the same age, but with distinct AM fungus colonization levels. We found that pea aphids show a reduced sE2 phase on the 84% AM fungus‐colonized plants, probably because of changes in food quality at this level of AM fungus colonization. This is supported by our data showing that the percentage of foliar N was significantly reduced in the 84% AM fungus‐colonized plants compared to the levels in the 42% AM fungus‐colonized plants (Table 2). These results are in agreement with previous reports showing that aphids spent more time in sieve elements of plants grown with N or high N than in sieve elements of N deficient or low N plants (Ponder *et al*., 2000; Nowak & Komor, 2010). In addition, aphids recognized the nutritional quality of the host plant primarily by the concentration of amino acids in the phloem sap, not by leaf surface cues nor by the quantity of essential amino acids (Nowak & Komor, 2010). Moreover, a decreased aphid population size was associated with reduced foliar N levels (Ponder *et al*., 2000; Bezemer *et al*., 2005). There are data showing that most ^15^N uptake by aphids occurs during feeding in the phloem phase E2 and the rest of the feeding activities do not contribute to N ingestion by aphids (Kuhlmann *et al*., 2013). However, it has been proposed that changes in aphid abundance on mycorrhizal plants are more related to changes in leaf morphology (e.g., phloem location and size) than to differences in foliar N content (Gange & West, 1994).

It is noteworthy that we found a significantly higher percent C in shoots of the 84% AM fungus‐colonized plants (43.7%) relative to control plants (40.5%) which might point out to thicker leaves since there were no differences in neither shoot fresh nor dry weight (Table 1). We found no statistical differences among treatments for phosphorus (P) and N/P (Table 2). Previous studies in *M. truncatula* showed significant increases in foliar P, percent C and N at 62 d postinoculation with *R. irregularis* (roots were about 80% total RLC) compared to nonmycorrhizal controls (Schweiger *et al*., 2014). Also, a significant increase in shoot total P content and shoot mass were reported at 72 d postinoculation with *G. versiforme* spores (roots were 44% and 40% RLC) compared to nonmycorrhizal controls (Javot *et al*., 2007). Based on previous data, it is not surprising that we did not detect a significant increase in total P content in shoots of mycorrhizal *M. truncatula* because plants were at 42–49 d postinoculation. However, a reduced percent N in the 84% AM fungus‐colonized plants (5.3% N) relative to the 42% AM fungus‐colonized plants (6% N) points to a poor food quality for aphids. In the present study, we detected changes at the plant tissue level related to the presence of AM fungi, but in order to reach additional conclusions, further studies would be necessary.

We also evaluated the percentage of aphids exhibiting specific EPG waveforms in 30‐min intervals during feeding for 8 h on *M. truncatula* with different levels of AM fungus colonization. We found that the percentage of aphids that showed phloem ingestion was significantly reduced during feeding on the 84% AM fungus‐colonized plants relative to the percentage of aphids on the 42% AM fungus‐colonized plants (23% vs. 73% of aphids in the 8th hour) (Fig. 2). Differences began to occur after the 4th hour of recording, showing that the number and duration of sE2 phase was significantly reduced when insects fed on the 84% AM fungus‐colonized plants (Fig. 3). It has been reported that mycorrhizal plants exhibit dramatic anatomical changes (increases in the thickness of leaves, size of midrib, major and minor veins, mesophyll cells, number of plastids, etc.) and increased levels of insoluble polysaccharides and proteins in leaves (Krishna *et al*., 1981), so we speculate that these changes could potentially have altered aphid feeding behavior in our study. Furthermore, it was shown recently that mycorrhizal wheat plants (26.7% RLC) had increased vascular bundle size, and it was proposed that this increase in size of the sieve element could be indicative of a healthier and more nutritious plant (Simon *et al*., 2017). Aphids were able to feed on insect‐resistant mycorrhizal plants, grow faster, and reproduce more successfully (Simon *et al*., 2017). Interestingly, *V. faba* plants inoculated early with AM fungi (∼40% RLC; colonized prior to aphid feeding) had 37% more pea aphids compared to control plants (Babikova *et al*., 2014a). This is in agreement with our results; we found that aphids that fed on the 42% AM fungus‐colonized plants were able to ingest phloem sap for a longer time compared to insects that fed on the 84% AM fungus‐colonized plants (WDI sE2; Table 3).

Surprisingly, not much is known about how aphids are affected when they encounter plants that are highly colonized by AM fungi. A model that explains mycorrhiza‐induced resistance (MIR) suggests that mycorrhizal plants exhibit priming, which results in quicker and more effective activation of JA‐dependent defenses (Pozo & Azcon‐Aguilar, 2007). Priming has also been demonstrated in the Arabidopsis–Rhizobacteria interaction with aphids, showing an enhanced expression of the JA‐responsive gene *LOX2* by the green peach aphid *M. persicae*, but not by the specialist aphid *Brevicoryne brassicae* L. (Pineda *et al*., 2012). Moreover, induced systemic resistance against *M. persicae* was activated by an endophytic bacterial strain *Bacillus velezensis* YC7010 (Harun‐Or‐Rashid *et al*., 2017). This model of MIR also explains why mycorrhizal plants show increased susceptibility to biotrophic pathogens and increased resistance to necrotrophic pathogens and generalist chewing insects (Pozo & Azcon‐Aguilar, 2007). However, in the case of phloem feeders (e.g., aphids) or specialist insects, these organisms benefit from feeding on mycorrhizal plants (Pozo & Azcon‐Aguilar, 2007). Some of the mycorrhiza‐induced changes related to plant defenses involve a transient increase in medicarpin levels in *M. truncatula* roots during the early stages of AM fungus colonization, but during the later stages of the interaction, medicarpin levels decrease (Harrison & Dixon, 1993). Hormonal levels, especially jasmonates, are also known to rise in *M. truncatula* and tomato (*S. lycopersicum*) roots during a fully established AM symbiosis (Hause *et al*., 2002; Lopez‐Raez *et al*., 2010). Nevertheless, few studies have examined the mycorrhiza‐induced systemic changes in shoots (Liu *et al*., 2007; Schweiger *et al*., 2014); most studies to date focused on local and systemic changes in roots. A study that examined transcriptome changes in both shoots and roots of mycorrhizal *M. truncatula* plants (39%–53% RLC) found that 26% of the genes induced in the shoots are predicted to be involved in abiotic or biotic stress signaling or responses (Liu *et al*., 2007). The data were further supported by the significantly reduced growth of the leaf pathogen *Xanthomonas campestris* on mycorrhizal plants compared to nonmycorrhizal plants (Liu *et al*., 2007). Moreover, it was reported that *M. truncatula* (80% RLC) was the most responsive plant species to AM fungi, showing that 14.7% of its metabolites were modulated in shoots (Schweiger *et al*., 2014). It was not until recently that plant gene expression was examined during aphid–plant–AM fungus interactions (Maurya *et al*., 2018). Genes strongly induced by aphid feeding in shoots (*β‐1,3 glucanase*, *thaumatin‐like protein*, *ethylene response factor (ERF) 1*, *gibberellin (GA) 20‐oxidase*, and *GA 2‐oxidase*), by AM symbiosis in roots (*oxo‐phytodienoic acid reductase*, *ERF1*, and *GA 20‐oxidase*), and during the three‐way interaction were identified (Maurya *et al*., 2018). It was found that pea aphid colony weight decreased or increased after 7 d of insect feeding on low (20%–40% RLC prefeeding) or high (>41% RLC prefeeding) AM fungus‐colonized *M. truncatula*, respectively (Maurya *et al*., 2018). Thus, the present study helps us better understand the impact of plant‐associated beneficial microbes on aphid feeding behavior, something that has been missing in previous studies, especially in legumes.

We consider that a single aphid should not be able to trigger such a strong plant defense response that could alter aphid feeding behavior, which is supported by data showing that induction of defenses in *M. truncatula* and *V. faba* is dependent on aphid density (Mai *et al*., 2014; Stewart *et al*., 2016). A pea aphid density of 0, 10, 25, 50, and 100 aphids per plant were used in *M. truncatula* to assess induction of defense hormones and the phytoalexin medicarpin. Medicarpin levels significantly increased with ≥50 aphids per plant, while abscisic acid and jasmonic acid (JA) levels were not significantly altered by aphid density. However, salicylic acid (SA) levels significantly increased with ≥25 aphids per plant (Stewart *et al*., 2016). In the present study, since we used only one aphid per plant, we predicted that any observed differences in feeding behavior should be the result of changes modulated by the AM symbiosis. Based on these data, we speculate that the 84% AM fungus‐colonized plants might exhibit MIR in shoots hindering the pea aphid ability to ingest phloem sap (Table 3; Figs. 2 and 3).

In conclusion, this study provides insight about how plant‐associated mutualistic microbes alter aphid‐feeding behavior on a leguminous plant. To our knowledge, this is the first report examining aphid‐feeding behavior on highly AM fungus‐colonized plants. Our key findings revealed that aphids needed more time to reach their 1st sE2 phase while feeding on the 84% AM fungus‐colonized plants compared to those insects feeding on the 42% AM fungus‐colonized plants or on control plants. Interestingly, we found that less aphids showed phloem ingestion (E2) during feeding on the 84% AM fungus‐colonized plants relative to the 42% AM fungus‐colonized plants. We propose that modifications in plant anatomy (e.g., thicker leaves), poor food quality (reduced N), and perhaps MIR in the 84% AM fungus‐colonized plants may have hindered the aphid's ability to find and reach the phloem leading to differences in phloem sap ingestion. Further research is still needed to understand the underlying molecular mechanisms involved in mediating resistance or susceptibility to aphids during insect–plant–microbe interactions.

## Disclosure

All the authors confirm that they have no financial or other involvement in activities or organizations that might bias the work reported here.

## Supporting information


**Fig. S1** (A) Schematic representation of the experimental set‐up. Only one of the eight possible probes is shown (figure provided by EPG‐Systems, Wageningen, the Netherlands). (B) Experimental set‐up showing *Medicago truncatula* plants and pea aphids (*Acyrthosiphon pisum)*. (C) Detail of gold wire attached to the pea aphid dorsum while feeding on a *M. truncatula* trifoliolate leaf.
