# Supplementary figures and images for: High levels of arbuscular mycorrhizal fungus colonization on Medicago truncatula reduces plant suitability as a host for pea aphids (Acyrthosiphon pisum)
Source: Insect Sci. 2018 Sep 17;27(1):99–112. doi: 10.1111/1744-7917.12631 (PMC7379733; doi:10.1111/1744-7917.12631)

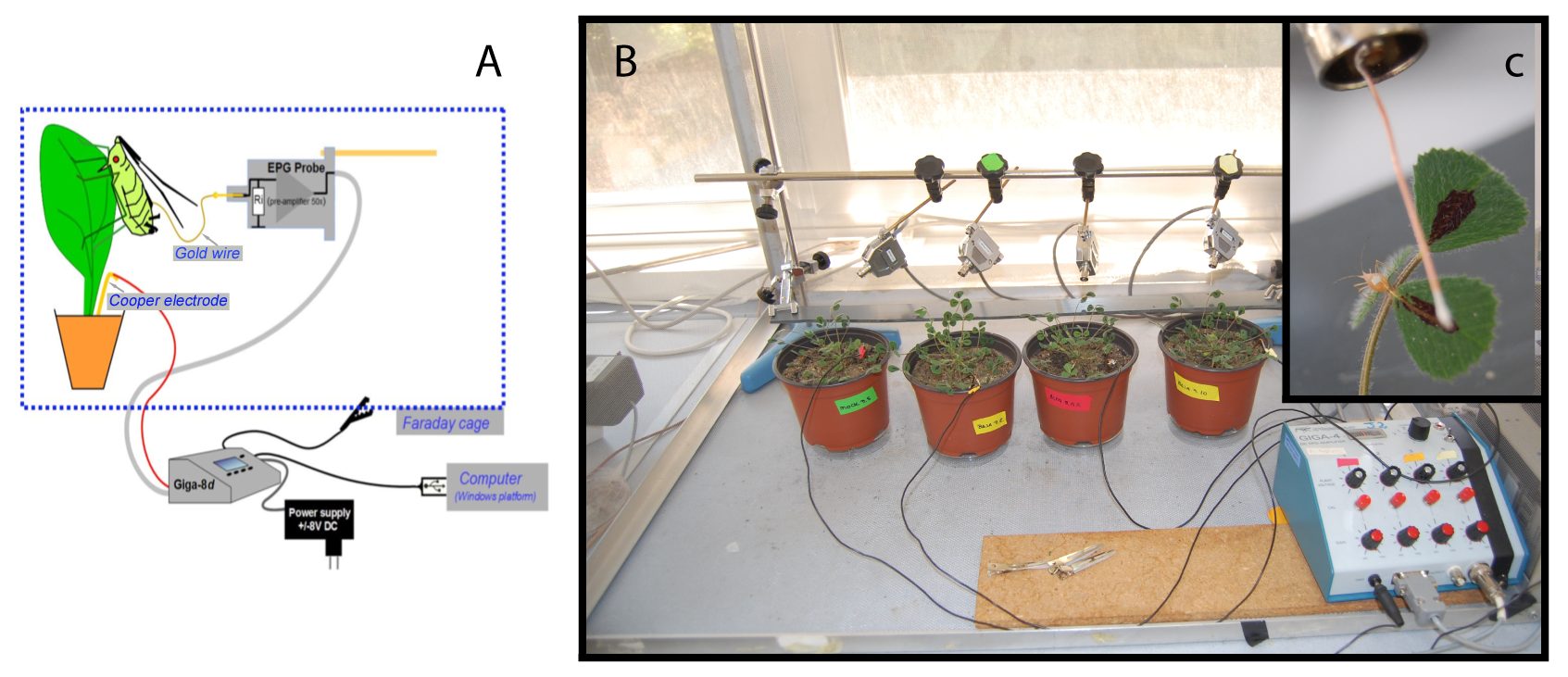

Supplement: Supplementary file 1 — Fig. S1 (A) Schematic representation of the experimental set‐up. Only one of the eight possible probes is shown (figure provided by EPG‐Systems, Wageningen, the Netherlands). (B) Experimental set‐up showing Medicago truncatula plants and pea aphids (Acyrthosiphon pisum). (C) Detail of gold wire attached to the pea aphid dorsum while feeding on a M. truncatula trifoliolate leaf. [file INS-27-99-s001.tif]
